# Sequence and vector shapes vaccine induced antibody effector functions in HIV vaccine trials

**DOI:** 10.1371/journal.ppat.1010016

**Published:** 2021-11-29

**Authors:** Stephanie Fischinger, Deniz Cizmeci, Davy Deng, Shannon P. Grant, Nicole Frahm, Julie McElrath, Jonathan Fuchs, Pierre-Alexandre Bart, Giuseppe Pantaleo, Michael Keefer, William O. Hahn, Nadine Rouphael, Gavin Churchyard, Zoe Moodie, Yeycy Donastorg, Hendrik Streeck, Galit Alter

**Affiliations:** 1 Ragon Institute of MGH, Harvard and MGH, Boston, Massachusetts, United States of America; 2 PhD Program in Immunology and Virology, University of Duisburg-Essen, Essen, Germany; 3 UC Berkeley, Department of Chemical and Biomolecular Engineering, Berkeley, California, United States of America; 4 Vaccine and Infectious Disease Division, Fred Hutchinson Cancer Research Center, Seattle, Washington, United States of America; 5 Bill & Melinda Gates Medical Research Institute, Cambridge, Massachusetts, United States of America; 6 Center for Learning and Innovation in Public Health at San Francisco Department of Public Health, San Francisco, California, United States of America; 7 University of California, Department of Medicine, San Francisco, California, United States of America; 8 Dept of Internal Medicine, Lausanne University, Lausanne, Switzerland; 9 Service of Immunology and Allergy, Department of Medicine, Lausanne University Hospital, University of Lausanne, Lausanne, Switzerland; 10 Swiss Vaccine Research Institute, Lausanne University Hospital, University of Lausanne, Lausanne, Switzerland; 11 Department of Medicine, University of Rochester, Rochester, New York, United States of America; 12 Department of Immunology, University of Washington, Seattle, Washington, United States of America; 13 Division of Allergy and Infectious Disease, Department of Medicine, University of Washington, Seattle, Washington, United States of America; 14 The Hope Clinic, Division of Infectious Diseases, Emory University School of Medicine, Atlanta, Georgia, United States of America; 15 Division of Infectious Diseases, Department of Internal Medicine, Emory University School of Medicine, Atlanta, Georgia, United States of America; 16 Aurum Institute, Parktown, South Africa; 17 School of Public Health, University of Witwatersrand, Johannesburg, South Africa; 18 Vaccine and Infectious Disease Division, Fred Hutchinson Cancer Research Center, Seattle, Washington, United States of America; 19 HIV Vaccine Trial Research Unit, Instituto Dermatologico y Cirugia de Piel (IDCP), Santo Domingo, Dominican Republic; 20 Institute of Virology, University Hospital, University of Bonn, Germany; 21 German Center for Infection Research (DZIF), partner site Bonn-Cologne, Germany; Emory University, UNITED STATES

## Abstract

Despite the advent of long-acting anti-retroviral therapy able to control and prevent infection, a preventative vaccine remains a global priority for the elimination of HIV. The moderately protective RV144 vaccine trial suggested functional IgG1 and IgG3 antibodies were a potential correlate of protection, but the RV144-inspired HVTN702 validation trial failed to demonstrate efficacy despite inducing targeted levels of IgG1/IgG3. Alterations in inserts, and antigens, adjuvant, and regimen also resulted in vaccine induced target quantitative levels of the immune correlates, but drove qualitative changes to the humoral immune response, pointing to the urgent need to define the influence of vaccine strategies on shaping antibody quality, not just quantity. Thus, defining how distinct prime/boost approaches tune long-lived functional antibodies represents an important goal in vaccine development. Here, we compared vaccine responses in Phase I and II studies in humans utilizing various combinations of DNA/vector, vector/vector and DNA/protein HIV vaccines. We found that adenoviral vector immunization, compared to pox-viral vectors, resulted in the most potent IgG1 and IgG3 responses, linked to highly functional antibody activity, including assisting NK cell related functions. Minimal differences were observed in the durability of the functional humoral immune response across vaccine regimens, except for antibody dependent phagocytic function, which persisted for longer periods in the DNA/rAd5 and rAd35/rAd5 regimen, likely driven by higher IgG1 levels. Collectively, these findings suggest adenoviral vectors drive superior antibody quality and durability that could inform future clinical vaccine studies.

**Trial registration**: ClinicalTrials.gov NCT00801697, NCT00961883, NCT02207920, NCT00125970, NCT02852005).

## Author summary

Previous HIV-1 vaccine trials, including the RV144 efficacy trial that resulted in moderate protection from infection, showed the importance of non-neutralizing antibody responses and their contribution to protection against the virus. However, so far it is unclear how to specifically induce these potentially protective responses via vaccination and the question remains which prime/boost vaccine strategy is the most promising one. In this study, we directly compared the humoral immune response elicited by different heterologous HIV-1 prime/boost vaccine regimen utilizing different components such as DNA, pox- and adenovectors (rAd) and protein. We found that the combination of a DNA or rAd prime with a rAd boost resulted in strong IgG1 and IgG3 responses, which have been shown to be associated with reduced risk in RV144. Moreover, these regimens induced long-lived ADCP responses, an antibody driven functional response mediated by monocytes, that has been shown to be protective in non-human primate trials. In the light of the ongoing efficacy trial HVTN 705 which is utilizing an adenovirus prime, this data could give important input to analyze Fc-mediated functions and non-neutralizing antibody titers in future efficacy trials.

## Introduction

Despite the development of highly effective, long-active anti-retroviral therapy (ART) that can control the Human Immunodeficiency Virus (HIV) [1] and even prevent infection if taken prior to exposure [2], 1.8 million individuals are newly infected with HIV every year [3]. Thus, a prophylactic vaccine against HIV remains critical in the ultimate control and elimination of HIV [4]. In the pursuit of an effective HIV vaccine, several vaccine immunogens, inserts, doses, vaccination schedules and routes of administration have been explored, with limited success. Using a pox-viral prime (ALVAC) and protein boost (AIDSVAX), the RV144 vaccine trial showed a modest level of protection, with 31.2% reduced risk of infection among vaccinated Thai individuals by 42 months [5,6]. While neutralizing antibodies were largely undetectable among vaccinees, immune correlates analyses highlighted an enrichment of ADCC and complement fixing IgG1 and IgG3 antibodies, directed at the 1^st^ and 2^nd^ variable loops (V1V2) of the envelope protein [7–10]. However, the follow-on HVTN702 trial, performed in South Africa using the same vector with a different insert, a different protein/adjuvant combination, and with an extended vaccine regimen, failed to confer any level of protection against infection [11]. It remains unclear whether qualitative, rather than quantitative, changes in antibody responses following these alterations in the HVTN702 regimen, compromised vaccine efficacy, highlighting our minimal understanding of vaccine platform/regimen effects on tuning antibody quality.

Notably, heterologous boosting in RV144 (ALVAC/AIDSVAX) induced higher IgG3 levels, linked to reduced risk of infection [12], compared to the preceding vaccine trial using the same protein vaccine antigen alone (VAX003) [9,13,14]. Conversely, the addition of a final boost in the RV305 trial, that extended upon RV144 with the addition of a final boost, eliminated these IgG3 responses [15], pointing to a critical role both for vector priming and boosting as critical determinants of antibody subclass selection. Several viral vectors have been shown to efficiently induce specific patterns of immunity, partially attributed to the co-delivery of pathogen-associated molecular patterns (PAMPs) [16,17] along with the antigen [18–20]. Specifically, Modified Vaccinia Ankara (MVA), nonreplicating attenuated poxvirus vectors (NYVAC and ALVAC), and several adenoviruses (Ad5, Ad26, Ad35, and Ad48) [21–24] have been tested, each driving distinct magnitudes of humoral and cellular immune responses, but how these vaccines tune the quality of the humoral immune response remains unclear. However, defining the specific humoral profiles induced by distinct vaccine strategies may provide critical clues for the rational design of vaccine strategies aimed at eliciting potential immunity to HIV or beyond.

Thus, to begin to define the impact of distinct heterologous prime/boost vaccine strategies on shaping vaccine induced humoral immune functional profiles and durability, we comprehensively profiled the vaccine induced immune responses in five distinct clinical HIV vaccine studies run by the HIV Vaccine Trials Network (HVTN). The trials included DNA-, vector- and protein-based regimens, in different combinations and sequences, allowing for the identification of immunogen-induced humoral signatures. Comparative analysis demonstrated significant differences both in the specificity and functional quality of antibodies induced by each prime/boost strategy and highlighted the importance of the addition of a DNA or adenoviral component to induce qualitatively superior functional antibodies. These data provide an unprecedented description of the antibody profiles induced by distinct vaccine platforms that could be used to more broadly inform vaccinology.

## Results

### Beyond IgG1 titers, subclass selection differs across vaccine approaches

**Fig 1:**
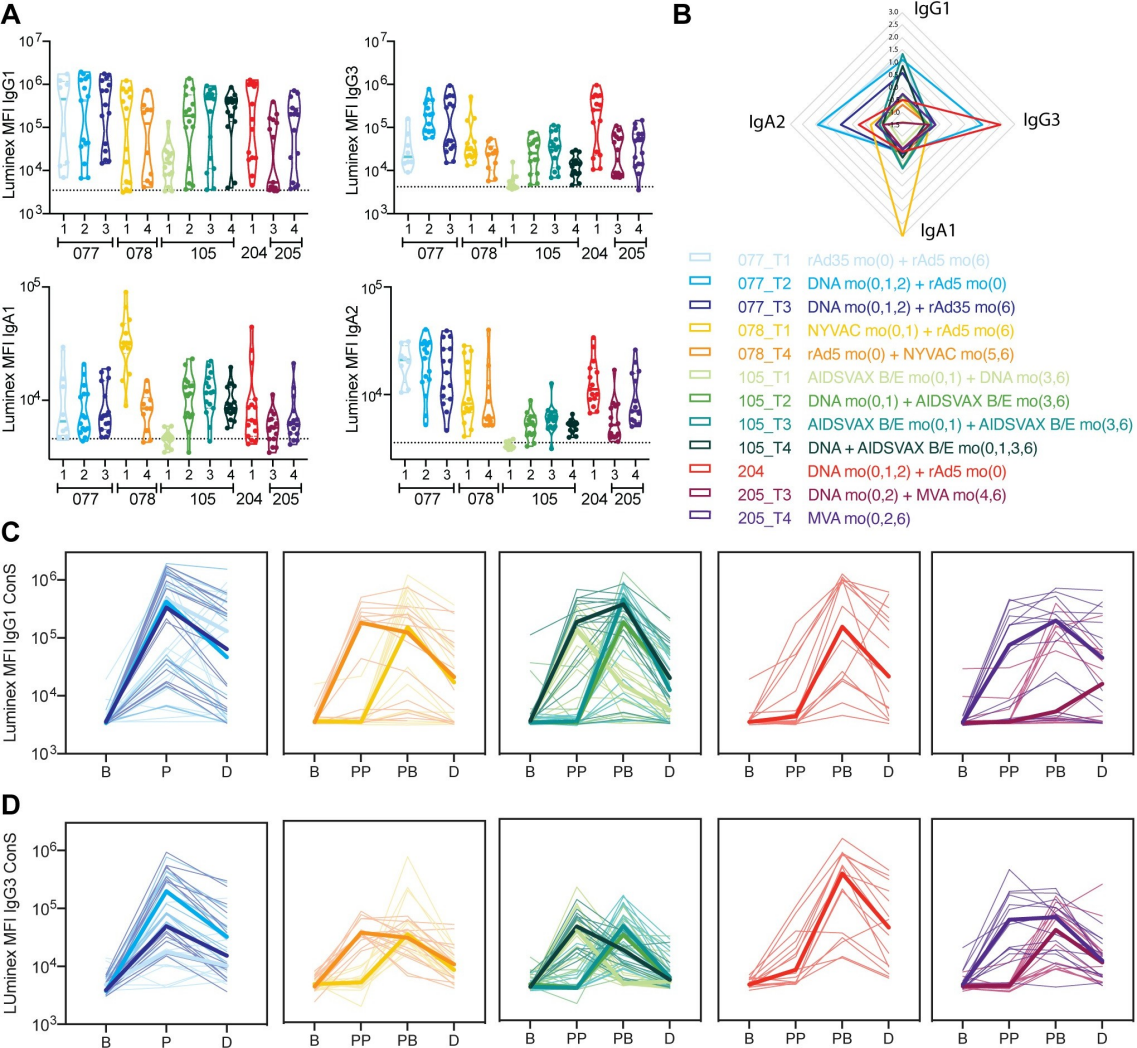
Subclasses/isotypes are tuned differentially across vaccine regimens. Plasma samples were assayed with a customized Luminex assay against gp140 ConS antigen and IgG1, IgG3, IgA1 and IgA2 relative titers in mean fluorescence intensity (MFI) are depicted here. **A:** The violin plot shows relative antibody titers for each vaccination arm for 5 HVTN trials at the peak immunogenicity time point, lines are indicating median and quartiles. The dotted line indicates placebo levels (averaged across all trials). IgG1, IgG3, IgA1 and IgA2 were measured against gp140 ConS. **B:** The radar plot shows combined IgG1, IgG3, IgA1 and IgA2 data, data for each subclass were z-scored. Colors correspond to the different trials as indicated in legend. **C-D:** Each line plot shows IgG1 (C) and IgG3 (D) levels for each trial over time starting at baseline (B), post prime (PP), post-boost (PB) and durability (D) time point. For trial 078, PP indicates Month 1, after the first but before the second prime administration. Different trial arms are depicted in different colors, placebo is indicated in black for panel A.

Previous immune correlates analyses in the RV144 vaccine trial pointed to an enrichment of vaccine-specific IgG1 and IgG3 responses in uninfected vaccinees [9], that were lost with repeated boosting in the VAX003 trial [14]. However, whether subclass/isotype selection is differentially tuned by distinct vaccine regimens remains unclear. Thus, initially the levels of IgG1, IgG3 and IgA responses were investigated across five different HVTN vaccine trials, at peak immunogenicity (Fig 1A and 1B) and 6 months post final immunization (Fig 1C and 1D). While all vaccine trials used different antigenic inserts, antibody titers were evaluated against a consensus gp140 sequence (ConS), to which all vaccinees responded and that represents diverse strains of Env [12,25].

Heterogeneity was observed in ConS IgG1 levels within arms and across the trials, with both strong responders and non-responders in each arm (Fig 1A). Similar overall levels of IgG1 were observed across most trials and arms, with the exception of the AIDSVAX/DNA trial 105 T1 that induced lower levels of IgG1 (all significances can be found in S1 Table). Analysis of IgG3 levels showed similar IgG3 levels within vaccine arms/groups (Fig 1A). However, significant differences were observed across vaccine arms/regimens. Trials employing a DNA prime with rAd5/rAd35 boost (077 T2 and 077 T3) and DNA prime/rAd5 boost (204) induced elevated IgG3 levels compared to other trials (Fig 1A and 1B). The AIDSVAX prime/DNA boost (105 T1), that induced lower IgG1, also induced low levels of IgG3. Conversely, several trials, including the rAd35 prime/rAd5 boost (077 T1), DNA prime/AIDSVAX boost (105 T2), AIDSVAX prime/AIDSVAX boost (105 T3), and DNA prime/AIDSVAX (105 T4) all induced low levels of IgG3 in the setting of high IgG1 levels.

While total IgA levels were defined as a correlate of risk in the RV144 vaccine trial, humans possess two IgA subclasses, IgA1 and IgA2, that are structurally distinct [26] and IgA has been implicated in protection in primate vaccine studies [27,28]. Surprisingly, IgA subclass levels varied significantly across vaccine arms/regimens (Fig 1A and 1B). IgA1 levels were enriched in the NYVAC prime/rAd5 boost (trial 78 T1) (Fig 1A and 1B). Conversely, higher levels of IgA2 responses were observed in all but the HVTN 105 trial arms AIDSVAX/DNA (T1), DNA/AIDSVAX (T2), AIDSVAX/AIDSVAX (T3) and DNA/AIDSVAX (T4), and the DNA/MVA (205 T3) arm, with the highest levels in the rAd35/rAd5 and DNA/rAd5 trials (Fig 1B). Thus, adenovirus including strategies all induce IgA2, but only NYVAC priming induced significant IgA1 levels.

We next profiled the kinetics of the vaccine induced immune responses (Fig 1C and 1D). Significant and expected differences were observed in the evolution of the IgG1 (Fig 1C) and the IgG3 (Fig 1D) responses across the studies. HVTN 077 T2 and T3, as well as 204, that all included a DNA prime followed by an adenoviral boost, induced comparable antibody kinetics, with robust induction of both IgG1 and IgG3 responses. In contrast, the kinetics of the humoral immune response differed for HVTN 077 T1, which applied a vector/vector strategy (rAd35/rAd5), and elicited slower kinetics, but that reached similar levels at the durability time point as those induced with the DNA primed vaccine regimens. Conversely, for HVTN 078, the administration of the rAd5 vector prime appeared to be essential in the early and robust induction of IgG1 responses, as peak antibody titers were observed after the Ad5 vaccination in both arms. Similarly, in HVTN 105, the administration of the AIDSVAX protein was essential in driving robust IgG1 titers, however a second protein boost or a heterologous immunization was required to elicit appreciable IgG3 responses, despite the rapid decline of both subclass responses over time. Finally, immunization with MVA only induced strong IgG1 and IgG3, whereas DNA priming was insufficient to drive these responses in the absence of the MVA boost in HVTN 205 T3 and T4, respectively. While IgG1 responses remained elevated in some MVA only vaccinees, both IgG1 and IgG3 responses declined rapidly in the heterologous immunization arm. These data collectively confirm the poor humoral inductive power of DNA alone and both the adenoviral and poxviral based regimens induced longer lived IgG1 responses over time compared to DNA/protein immunization.

### DNA/rAd and rAd35/rAd5 vaccination drive high functionality

**Fig 2:**
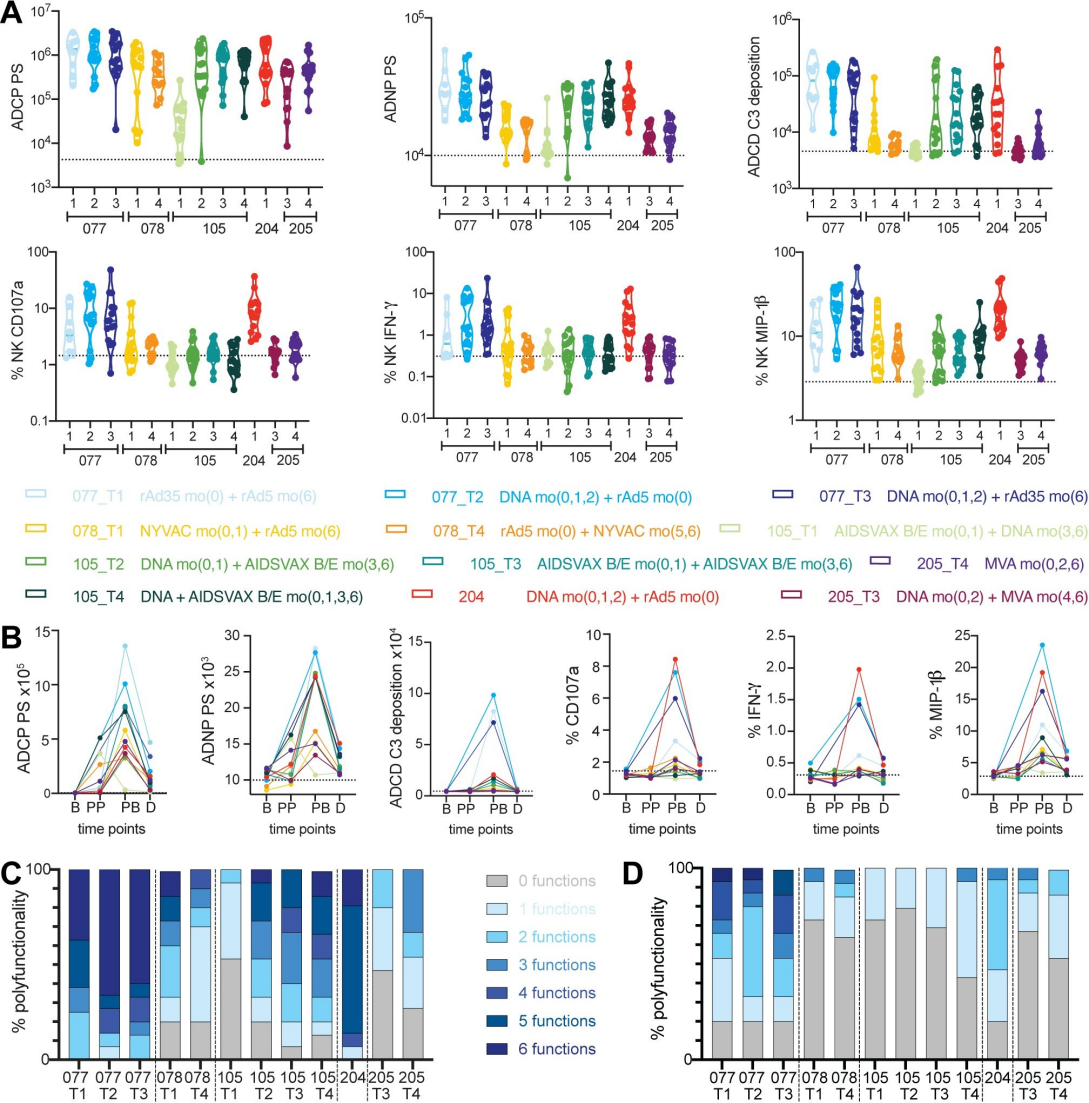
Vaccine regimens drive distinct antibody functional profiles. **A:** The violin plots depict six functional assays which were performed against a gp140 ConS antigen at peak immunogenicity (Month 6.5/7 post vaccination initiation), lines are indicating median and quartiles. The different trials and trial arms are depicted along the x-axis, each trial arm is shown in a different color, placebo levels are represented by the dotted line. **B:** Each line graph depicts one effector function over time for each trial arm. Time points include baseline (B), post prime (PP), post-boost (PB) and durability (D). For trial 078, PP indicates Month 1, after the first but before the second prime administration. The lines represent the median of each trial arm, with each trial arm depicted in a different color. The stacked bar graphs show the polyfunctional profile across all trial arms at peak immunogenicity (**C**) or the durability time point (month 12) (**D**), calculated as the number of functions within the group above the overall median across the whole cohort for each function. Colors indicate the polyfunctionality from light (0,1,2 functions) to dark (more polyfunctional).

In order to further dissect the humoral immune response across vaccine strategies, the ability of antibodies to recruit innate effector functions including antibody-dependent cellular phagocytosis (ADCP), antibody dependent neutrophil phagocytosis (ADNP), antibody dependent complement deposition (ADCD) and antibody dependent NK cell activation (degranulation: CD107a and cytokine/chemokine secretion: IFN-γ and MIP-1β) was assessed. These assays were conducted against the gp140 ConS antigen at the peak immunogenicity (Fig 2A) (all significances are listed in S1 Table).

ADCP activity was observed across most trial arms, other than the AIDSVAX/DNA trial 105 T1 arm (Fig 2A). In contrast, trial arms with AIDSVAX only (105 T3) or DNA prime/AIDSVAX (105 T4) and DNA/rAd5 and rAd35/rAd5 in HVTN 077, induced the highest levels of ADCP. Very few functional differences between strategies with an rAd5 or rAd35 boost were observed, indicating that both serotypes induce similar levels of functionality, regardless of the changes in vaccine schedule with rAd5/rAd35 boost being administered at month 6.

ADNP and ADCD generally demonstrated similar profiles within a particular regimen but we observed interesting differences across regimens. In contrast to ADCP which was induced by most regimens, the NYVAC/Ad5 arm (078 T1), the protein/DNA regimen in 105 T1, as well as the MVA only arm in 205 all induced negligible ADNP and ADCD levels despite the induction of detectable IgG1 responses (Fig 1A). On the other hand, the trials involving a DNA prime and an adenovirus boost, as well as rAd35/rAd5 regimen, induced high levels of ADCP, ADNP and ADCD, indicating that these vaccine regimens induced optimized polyfunctional antibody profiles. DNA prime/protein boost and trials with protein alone also induced all three functions, but to a slightly lower degree.

Strikingly, despite consistent IgG1 levels across the studies (Fig 1), only HVTN 077 and 204 induced NK degranulation and IFN-γ and MIP-1β secretion (Fig 2A). These same trials that also elicited higher levels of IgG3. These data suggest that DNA/adenovirus strategies, as well as to a lower degree rAd35/rAd5 vaccination, were able to induce higher levels of IgG3 responses, which are key to successfully leveraging NK cell functionality. Thus, significant functional differences were observed across studies, where NK cell function was largely observed in the setting of IgG3 inducing regimens, but ADCP/ADNP/ADCD were differentially induced in vaccine arms, disconnected from the overall vaccine induced IgG1/IgG3 levels. These data suggest that other modifications, such as post-translational modifications, may be responsible for the differences in antibody functionality we observed with ADCP/ADNP/ADCD.

Despite the significant differences functional profiles at peak immunogenicity across the vaccine arms, we next examined the durability of the functional response at later timepoints. All antibody effector functions declined significantly from peak immunogenicity, with limited but above baseline levels persisting for ADCP, ADNP, and NK cell mediated MIP-1β secretion (Fig 2B), largely among DNA/rAd containing regimens (HVTN 077 and 204). Thus, the decline in antibody function were more pronounced than the decay of overall antibody IgG titers across groups (Fig 1C and 1D), albeit the functional preservation was observed in the vaccine groups that maintained the highest titers.

Beyond single functional profiles, comparison of vaccine induced profiles in RV144 versus AIDSVAX vaccinees highlighted the presence of functional coordination among vaccine induced responses in the RV144 vaccine trial [14]. Thus, we next examined the overall polyfunctional humoral immune response across the different vaccine groups both at peak immunogenicity (Fig 2C) and at the durability timepoint (Fig 2D). DNA/rAd immunization groups (HVTN 077 and 204) exhibited the greatest polyfunctionality at peak immunogenicity, followed closely by DNA/AIDSVAX protein immunization (HVTN 105). Conversely, despite comparable vaccine induced titers, NYVAC/rAd5 and DNA/MVA immunization (HVTN 078 and 205) induced more modest polyfunctionality profiles at peak immunogenicity. Similarly, DNA/rAd5 regimens (HVTN 077 and 204) maintained moderate polyfunctional profiles one year after final vaccination, whereas all other arms exhibited minimal polyfunctionality linked to a loss of antibody effector function (Fig 2B).

### DNA/rAd5 and rAd35/rAd5 regimens elicit highly coordinated functional responses

**Fig 3:**
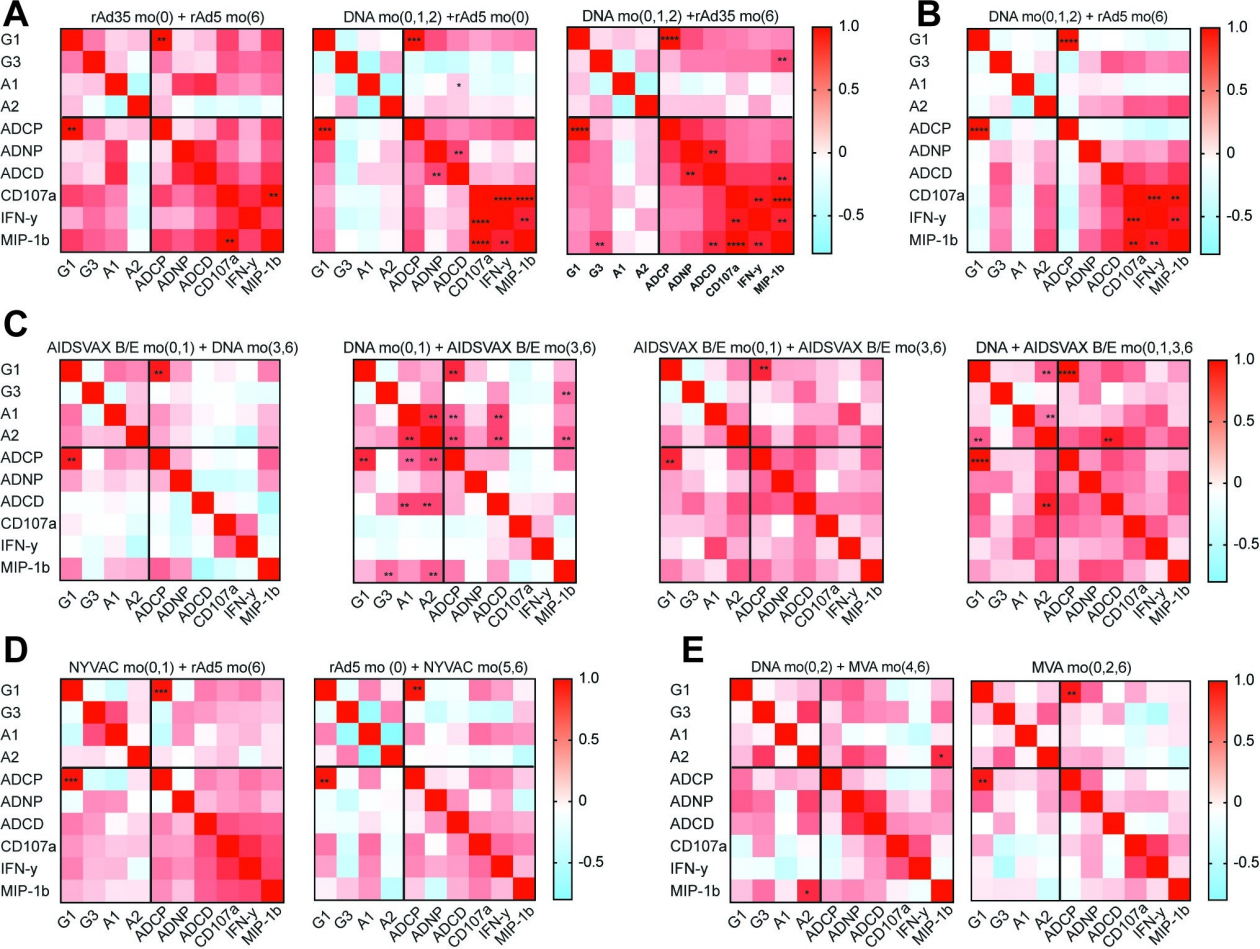
Subclass/isotype functional coordination varies by regimen/vaccine strategy. The heatmaps show Spearman correlations across antibody functional measurements and subclass/isotype levels for: **A:** 077 trials, with each heatmap depicting a different trial arm, left to right: T1, T2, T3; **B:** the DNA and rAd5 trial 204; **C:** the DNA/AIDSVAX B/E trial 105, trial arms T1 to T4; **D:** Trial 078 NYVAC/rAd5 (T1 and T2 arms); **E:** Trial 205 using DNA/MVA (T3 and T4). Red depicts high positive correlation coefficients, whereas blue displays a negative correlation, P values were Bonferroni corrected for multiple comparisons, *p<0.05, **p<0.01, ***p<0.001, ****p<0.0001.

Beyond univariate differences in subclass/isotypes and functions, we next aimed to determine whether the quality of the coordination in the humoral immune responses differed by vaccine regimen/sequence. Correlation heatmaps were generated for each vaccine regimen (Fig 3), highlighting the relationships within IgG1, IgG3 and IgA responses (left top), between isotypes/subclasses and functions (left bottom), and within functions alone (right bottom). Heterogeneous relationships were observed across the trials, with some arms showing robust functional coordination (e.g., HVTN 077 T1-T3, rAd35/rAd5 or DNA/rAd35) (Fig 3A) and others showed little to no coordination in humoral immunity (HVTN 105 T1, AIDSVAX+DNA) (Fig 3C). In general, most rAd including regimens (Fig 3A and 3B) showed strong functional coordination consistent with the robust polyfunctional profile observed within these arms (Fig 2C). An interesting pattern emerged in the rAd5/NYVAC trial (Fig 3D), where priming with NYVAC, followed by a rAd5 boost induced a more coordinated response than using a rAd5 prime and NYVAC boost (Fig 3D), highlighting the potential importance of sequence of vaccination. Finally, despite the induction of higher titers of antibodies with MVA immunization alone (Fig 1A), a DNA prime followed by an MVA boost seemed to induce a more coordinated humoral immune response (Fig 3E), arguing for the importance of heterologous prime/boosting as a means to improve antibody functionality. However, while positive relationships were observed across several arms/regimens, only rAd incorporating regimens showed significant functional coordination, independent of titer (Fig 1) which were induced at a high level across several arms.

To dissect the titer independent influences on functional coordination, we next examined relationships of the functions to antibody subclass/isotype levels. Variable relationships were observed across isotypes/subclasses and functions. IgG1 levels were positively correlated to most functions in most vaccines. IgG3 levels were variably and inconsistently associated with functions, other than some evidence of a relationship between IgG3 and NK cell MIP-1β secretion in HVTN 077 T2 (DNA/rAd35) (Fig 3A). Similarly, IgA levels were also variably associated with functions, including positive significant relationships for DNA/AIDSVAX (HVTN 105 T3 and T4) (Fig 3C). In the trials with low functional coordination (HVTN 105, 078 and 205), limited relationships were observed beyond the relationship between IgG1 and ADCP. These data point to heterogeneous subclasses/isotype functional coordination profiles across vaccine regimens, pointing to the importance of vaccine composition and regimens in leveraging IgG but also IgA bioactivity. However, it is important to note that these correlated responses were measured against a consensus antigen and not the actual immunogen, therefore, these measurements may not assess all nuances of vaccine induced immune variation, but provide an approximation of the response to a single clade c antigen.

### Distinct overall humoral profiles exist across trials

**Fig 4:**
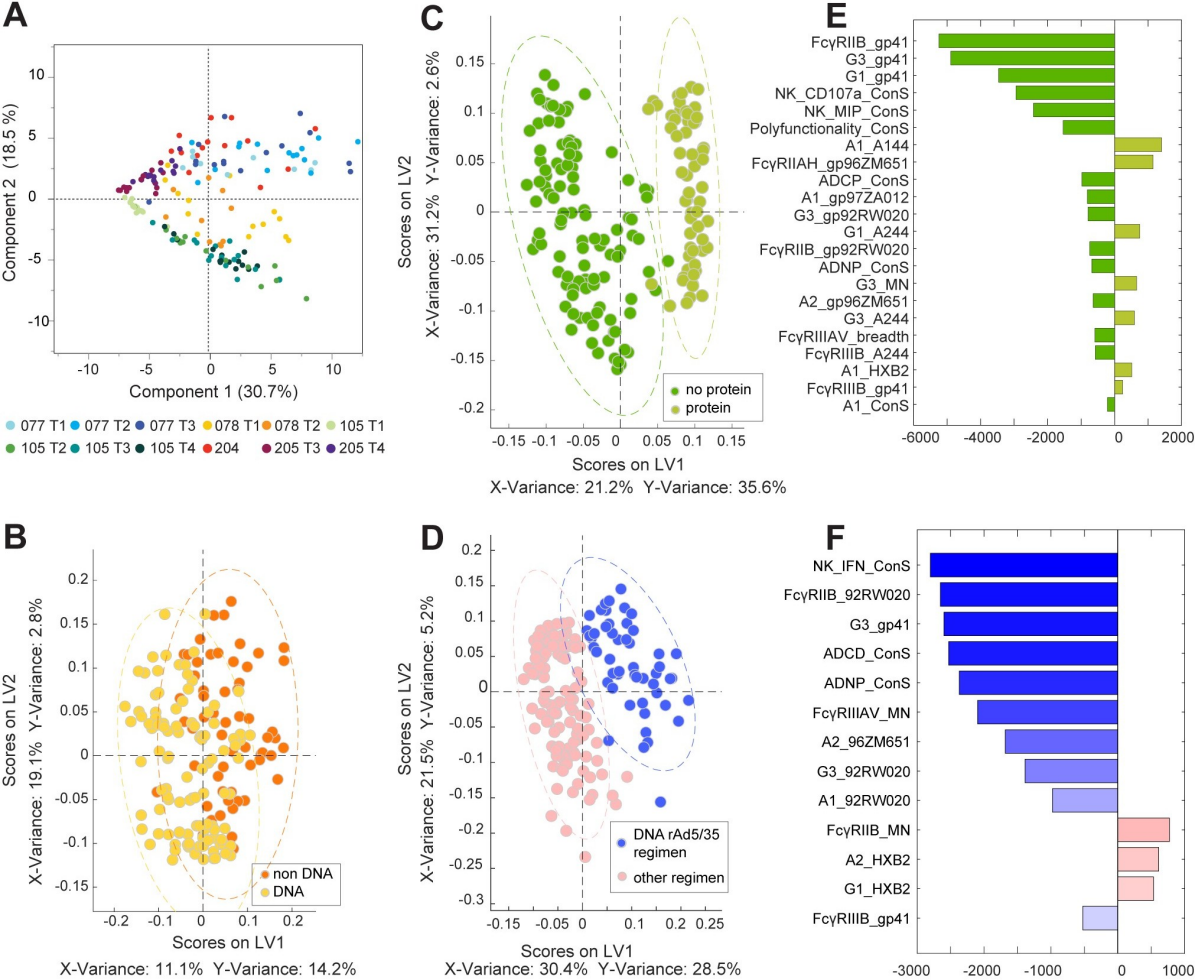
DNA and rAd5 including regimens induce distinct antibody profiles. Multivariate analysis of antibody profiles using 69 features for each plasma sample was performed. **A:** An unsupervised principal component analysis (PCA) was first used and demonstrates the separation of the different vaccine trials based on their antibody profiles. Each dot represents one vaccinated individual at peak immunogenicity and the colors indicate the trial arm as depicted in the legend. Next using a supervised PLSDA, differences in antibody profiles was examined in: **B:** DNA containing regimens (HVTN 077 T2, T3; 105 T1, T2, T3; 204; 205 T3) versus trials which do not incorporate DNA in their vaccine regimen (HVTN 077 T1; 078; 105 T3; 205 T4)- with a model accuracy of 77.3%; **C:** protein containing regimens (HVTN 105 all arms) compared to non-protein containing immunizations (HVTN 077, 078, 204, 205, all arms), with a model accuracy of 75.8%; and **D:** DNA/rAd containing regimens (HVTN 077 T2, T3; 204) versus all other regimens (HVTN 078, 105, 205) -with a model accuracy of 90.7%. **E** and **F** show the antibody features that were differed most significantly across the vaccine groups, ranked based on their Variable in Projection Score (VIP). Features are color coded based on the vaccine group in which they were enriched. **E:** variables pointing to the left (dark green) are enriched in vaccines without a protein component whereas features pointing to the right are increased in protein vaccine trials (light green). **F:** Features that point to the left were enriched in DNA + rAd5/rAd35 trials (blue), while those ordered on the right side are enhanced in other vaccine regimen trials (pink).

While univariate biophysical and functional differences already displayed discrete differences across vaccine profiles, we next aimed to define common differences across vaccine strategies (Fig 4A). A principal component analysis (PCA), capturing 69 antigen-specific antibody parameters per plasma sample, demonstrated the clear differences in vaccine induced antibody profiles, with nearly perfect separation across each vaccine regimen using this unsupervised method. Interestingly, a dominant split occurred using a protein boost versus vectored immunization, highlighting the importance of vaccine strategy in shaping vaccine induced Fc-profiles. Specifically, vaccine regimens that induced the lowest antibody titers (HVTN 205 MVA and 105 AIDSVAX/DNA) clustered together on the left side of the plot. Conversely, trials with higher titers and functions spread away from the low titer profiles. All three remaining protein arms, including DNA prime/AIDSVAX boost (HVTN 105 T2), AIDSVAX administration (105 T3) and DNA/AIDSVAX prime and boost (105 T4), clustered together, indicating that if a protein boost was administered, similar antibody responses were elicited. In contrast, vaccine regimens including NYVAC (HVTN 078), were distributed between the protein vaccine (bottom) and DNA/adenovirus vaccine (top) profiles, suggesting that NYVAC prime/boosting induced different responses (Fig 4A). However, whether NYVAC was used as a prime or boost did not differentiate antibody profiles in the PCA. The other vector/vector vaccine (HVTN 077 T1), including both rAd35 and rAd5, induced different antibody Fc-profiles, clustering with the vaccine regimen that included a DNA prime with rAd5/rAd35 (HVTN 077 T2, T3 and 204), indicating that rAd dominated the functional characteristic of vaccine induced antibodies.

Since we had observed striking differences in trials using protein, DNA and vector immunogens in the PCA analysis. Thus, we next aimed to define how each of these platforms shaped the vaccine induced Fc-profile of vaccine induced antibodies. All vaccinees were integrated and grouped by DNA versus no DNA (Fig 4B), protein versus no protein (Fig 4C), or adenovirus versus no adenovirus (Fig 4E). A supervised Partial Least Square Discriminant Analysis (PLSDA) was used to then define whether the profiles were distinct in each comparison, and then for analyses that showed separation between the arms, the specific antibody profile features that were most dramatically altered were plotted using a variable importance in projection (VIP) score (Fig 4D and 4F), that ranked each feature based on how differentially it was expressed across the two groups. Limited separation was observed in antibody profiles across individuals that received a DNA vaccine compared to those that did not (Fig 4B), suggesting that DNA immunization is unlikely to be a major driver of changes in vaccine induced antibody Fc-profiles. Conversely, robust separation was observed across the protein (Fig 4C) and rAd-immunized samples (Fig 4D).

Given that the AIDSVAX reagent lacks gp41, the dominant difference across protein immunized individuals was a lack of gp41-specific humoral immunity (Fig 4D). Additionally, protein immunized individuals also developed lower NK cell recruiting antibodies and lower polyfunctionality. Conversely, protein boosted individuals gained IgA responses, enhanced Fcγ-receptor IIa binding and IgG3 responses to additional antigens (Fig 4C). This indicates that while protein boosting has been shown to induce high titers [29], in combination with a DNA prime and other regimens, trials not including protein elicited a more polyfunctional, IgG1 and IgG3 driven profile.

Moreover, PLSDA of rAd containing regimens (blue) displayed significantly different antibody Fc-profiles compared to non-rAd containing trials (pink) (Fig 4E). Strikingly, rAd including regimens induced enhanced IgG1, IgG3, IgA, ADCD and ADNP activity, suggesting that this strategy is able to induce a highly diverse functional profile. Thus, supervised vaccine profile analysis demonstrates the presence of some common Fc-profiles that are selectively tuned using protein- or rAd vector- based immunization approaches.

### Polyfunctionality is driven by IgG1 titers and differential Fc-receptor binding

**Fig 5:**
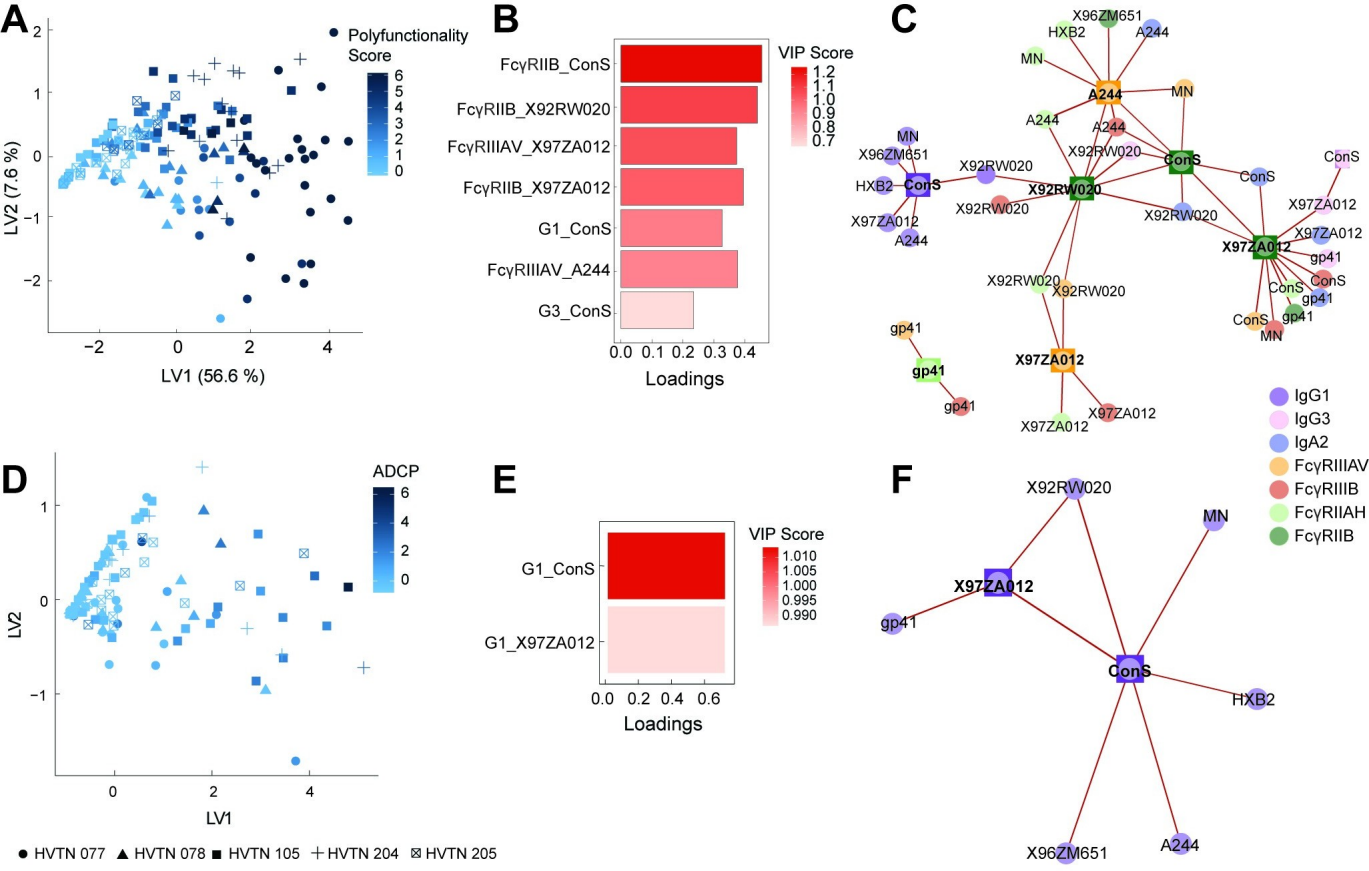
IgG1 and Fcγ-receptor binding profiles at peak immunogenicity track with polyfunctionality and durability. **A:** A Partial Least Squares Regression analysis (PLSR) shows the regression of all antibody profile features (72 features) on polyfunctionality for all the trials at peak immunogenicity. Trials are indicated by different shapes. The degree of polyfunctionality is depicted by the blue shade. **B:** The Loadings plot shows variables associated with polyfunctionality, loadings were scaled, and the color indicates the VIP score, VIP scores >0.7 are shown. **C:** The network shows the features associated with the selected variables from the Loadings plot which are drawn as rectangles, whereas co-correlated features are depicted as circles. Colors indicate the type of measurement as indicated in the legend. A Spearman correlation was performed, and only features with r>0.7 and p<0.01 after Benjamini-Hochberg correction were included. **D:** The PLSR plot shows the regression of all antibody features (72 features) at peak immunogenicity and ADCP levels at the durability time point (12 months). The shape depicts the trial. The shade of blue indicates the ADCP phagoscore, which was z-scored across all trial responses. **E:** The Loadings plot shows the only 2 critical features at peak immunogenicity associated with ADCP levels at the durability time point, colors indicate the Z-score and loadings were scaled. **F:** The network shows the co-correlates of peak immunogenicity predictors of ADCP durability. Squares depict the PLSR selected features and circles depict the co-correlates, while the color indicates the type of measurement. A Spearman correlation was performed to select the co-correlates, only features with r>0.7 and p<0.01 were included.

Within a polyclonal pool of antibodies, multiple antibody subclasses/isotypes may contribute to the induction of antibody effector functions. Moreover, given the different relationships between IgG1, IgG3, and IgAs with antibody effector functions across the different vaccine strategies (Fig 3), a partial least square regression (PLSR) analysis was used to define the multivariate antibody features associated with this highest level of polyfunctionality across all trials (Fig 5A). Thus, all Fc-features were regressed onto polyfunctionality (Fig 5A). Striking separation was observed across individuals with high polyfunctionality (dark blue) compared to those with non-polyfunctional responses (light blue). The variables that separated these profiles were then ranked in a VIP plot, with Fc receptor (FcγR) binding antibodies to the vaccine antigen at the very top of the profile, followed closely by IgG1 and IgG3 responses (Fig 5B). The selection of FcγRIIB binding antibodies as the top feature, the sole inhibitory FcγR in humans, was unusual and unexpected. However, FcγRIIB binding antibodies were tightly correlated to nearly all IgG1 titers as well as antibody binding to nearly all low affinity FcγRs and IgG3 levels (Fig 5C). These data suggest that the elicitation of poly-FcγR binding antibodies, rather than total level of IgG1 and IgG3 antibodies alone, is an essential correlate of polyfunctional immunity. Moreover, while IgG3 is regarded as the most functional antibody subclass in humans, the importance of IgG3 is clearly outweighed by highly functional IgG1-responses across these vaccine regimens.

### Durability of ADCP is dependent on peak IgG1 levels

In addition to defining the specific drivers of enhanced polyfunctionality, we finally aimed to define the specific vaccine induced profiles that were associated with durability across the vaccine trials. Whereas most functions showed a drastic decline in functional levels over time post final immunization (Fig 2B), ADCP levels persisted in some regimens (month 12) above background levels. Given the importance of improving durability in any prospective HIV vaccine, we examined the peak immunogenicity profiles that were associated with maintained ADCP activity (Fig 5D). Further dissection of the co-correlates at peak immunogenicity that tracked with persistent ADCP activity highlighted the presence of a single network, formed largely by IgG1 features across antigens, pointing to the induction of robust IgG1 responses as a key predictor of durable ADCP immunity (Fig 5F). Moreover, high levels of gp41-specific IgG1 responses at peak immunogenicity were a predictor of maintained functionality. Few FcγR-binding antibody features were also included in the network, including binding to FcγRIIB and FcγRIIIA and B, highlighting the importance of particular functional qualities at peak immunogenicity as a predictor of durable functionality. Overall, these findings indicate that induction of broad binding IgG titers with some level of FcγR binding rather than the induction of additional subclass/isotype levels is a critical predictor of durable antibody functionality, largely driven by IgG1 responses that are preserved over time (Fig 2).

## Discussion

Emerging data from vaccine development efforts across multiple pathogens suggest that understanding variations in prime/boosting strategies may not only be essential to expand B-cell memory, but to also drive longer lived high titer responses, and condition life-long vaccine immunity [5,30]. Specifically, while homologous prime-boosting regimens are typically administered for most clinically approved vaccines, heterologous prime-boosting has been shown to be more immunogenic, resulting in enhanced antibody titers in both mice [31] and non-human primates [32]. Linked to our growing appreciation for the role of adjuvants and early inflammatory events in shaping antibody effector profiles, understanding how prime-boosting strategies affect antibody effector function offers a unique opportunity to begin to rationally choose select combinations to selectively drive protective immune profiles of interest.

Several vaccine combinations have been explored in the context of HIV vaccine development, including DNA/vector, DNA/protein and vector/protein combinations. Thus, here we elected to begin to profile the functional humoral impact of combinatorial changes in prime/boost strategies across a number of HVTN HIV-1 Phase I and II trials using Systems Serology [33]. Overall, strategies with a DNA or rAd prime followed by either a rAd or protein boost showed the highest antibody titer and functional responses. However, all responses declined rapidly post the last boost, except for ADCP, which was maintained for longer periods of time.

Aside from the composition of a vaccine regimen, route of vaccine administration, vaccine dosage, interval between boosts, and adjuvants have all be implicated in shaping vaccine induced immunity [29]. The data shown here clearly suggest that that the vaccine response was influenced strongly by the choice of immunogen and insert, and further future dissection of the functional response to the unique vaccine-insert may highlight further differences across vaccine induced immune responses. However, the comparison across all vaccines against a consensus gp140 sequence allowed for a harmonized analysis of antibody functional programming, and provides a picture of the immune profiles against a particular challenge scenario. Yet, population level HLA-variation and pre-existing immunity are also likely to shift overall immune profiles within vaccine strategies [34,35]. Along these lines, significant variation was observed in IgG1 levels both within arms as well as across arms (Fig 1A). Conversely, IgG3 were more homogeneous within trial arms, but differed significantly across vaccine regimen, with robust IgG3 levels induced in the presence of DNA/Ad35 or DNA/Ad5 immunization. Analogously, enhanced antibody effector functions, and particularly NK cell function, were noted in the groups that received this sequence of DNA/rAd, likely related to the induction of robust IgG3 levels. Interestingly, IgA1 levels were induced following NYVAC priming and low level IgA1 were noted in the absence of a vectored vaccine strategy. These data suggest that viral vector tropism and specific inflammatory cascades triggered at the time of immunization [36], delivered by the vector themselves, is likely to be key to shaping subclass/isotype selection and therefore antibody effector function.

Vaccination with a DNA prime and rAd or protein boost induced the highest polyfunctionality, at both peak immunogenicity and durability time points (Fig 2C and 2D). Regimens that induced a polyfunctional response also induced more coordinated functional responses, including a balanced IgG1 and IgG3 profile (Fig 3)[14,33] and broad Fcγ-receptor binding profiles (Fig 5B). Interestingly, among the top predictors of polyfunctionality was the ability to bind to specific Fc-receptors, including FcγRIIB and FcγRIIIA, involved in phagocytosis and NK cell activation, respectively. While FcγRIIB is the sole inhibitory Fc-receptor in humans, it was linked to binding to multiple additional activating Fc-receptors (FcγRIIA, IIIA, IIIB) as well as to IgG3 levels. Likewise, FcγRIIIA binding predictors of polyfunctionality were linked to broad FcγR binding, highlighting the importance of FcγR binding profiles, and not solely IgG titers, as determinants of higher antibody effector function. Given our appreciation for the potential importance of adjuvants and inflammatory signals in shaping antibody Fc-glycosylation [37,38], these data suggest that vaccines able to tune the qualitative (subclass and glycosylation) profile of vaccine-induced antibodies, irrespective of antibody titer, may drive more polyfunctional antibodies.

While most trials induced strong titers of IgG1 and partly IgG3 at the peak immunogenicity, differences in the kinetics of antibody decline occurred after the last boost, driven by the peak titer and regimen. While DNA/rAd or AIDSVAX strategies induced the highest IgG1 titers following the last boost, titers declined rapidly. Vaccination with DNA/MVA or MVA however, while inducing lower levels of IgG1 at the peak time point, showed a less steep decrease after the last boost [39]. For IgG3 titers, trials that induced higher titers at peak immunogenicity also maintained higher levels over time, with significant differences between high/low peak titer inducing regimens. This suggests that while DNA/rAd5 strategies induced the highest IgG1 and IgG3 titers, MVA strategies may generate longer-lived immune responses. However, despite the rapid waning, the induction of high level IgG3 by DNA/rAd5 may be desirable, as described in a NHP study comparing the DNA/rAd5 strategy with a DNA/MVA vaccination. This strategy resulted in elevated titers and more protective immune responses following rAd5 boosting compared to MVA boosting [40], suggesting that a high initial response that wanes faster post boost may still be favorable.

ADCP was the only function that persisted at the durability timepoint (Fig 2B). ADCP has been linked to protection against SIV/SHIV infection in non-human primates [41]. Additionally, ADCP has been implicated in protection against malaria [42] and SARS-CoV-2 infection [43]. Moreover, analysis of the failed HVTN 505 trial, using a DNA/rAd5 vaccine, showed enhanced levels of ADCP in protected vaccinees and an association with reduced risk of infection [12]. Why ADCP persists while complement and NK cell functions decline is unclear. However, it is likely that particular inflammatory IgG-glycosylated populations may decline more rapidly than others. Thus, despite the persistence of IgGs over time, qualitatively different subpopulations of IgGs may persist for variable amounts of time. Furthermore, differences in intracellular signaling depending on cell type and FcγR activation, as well as IgA interference [44] could lead to differential persistence of ADCP. Thus, further investigation into the specific decay kinetics of distinct IgG subpopulations as well as their differential inducibility by distinct vaccine strategies may provide further insights to rationally design functional vaccine regimens able to persist for longer periods of time.

The recently terminated clinical efficacy trial, HVTN 702, highlighted the inability of the original RV144 vaccine regimen to confer protection in the context of a different viral clade and distinct risk group [11]. These data suggest that distinct immunization strategies may be required to confer protection in a high-risk setting. While Ad26-vaccine platforms have shown some level of protection in primates [41,45], results from HVTN 705 and MOSAICO have yet to be released. In the meantime, emerging data point to some level of success on the path to eliciting broadly protective neutralizing antibodies [46]. However, it is likely that a combination of neutralization and Fc-effector function are likely to be key to maximal protective immunity against HIV. Thus, the data presented here point to the possibility that particular prime/boost strategies may drive more robust and functional humoral immune profiles, compared to those induced with the RV144 regimen alone. Yet, future research able to compare Ad26 vector-induced immunity to RV144 induced immunity could provide additional critical insights. With the looming opportunity to compare immunogenicity in the HVTN702 and HVTN705 trials, linked to efficacy, this comparison, in a real-world setting is on the horizon. Thus, as our appreciation for additional protective antibody functions across pathogens expands [42,47,48], the ability to define “flavors” of humoral immune responses that are elicitable by distinct vaccine platforms, provides novel insights for the rational design of next generation vaccines against HIV and beyond.

## Methods

### Ethics statement

All subjects gave their informed consent for inclusion before they participated in the studies. The study was conducted in accordance with the Declaration of Helsinki, and the secondary use of specimen for Systems Serology was approved by the Ethics Committee (Project identification code 2017P001320).

### Samples

Plasma samples were provided by the HVTN (ClinicalTrials.gov Identifier: NCT00801697, NCT00961883, NCT02207920, NCT00125970, NCT02852005). All participants provided written informed consent, and the study was approved by the Institutional Review Board of Massachusetts General Hospital, IRB approval # 2017P001320. Vaccination schedule and regimen for each trial and each trial arm is depicted in Table 1. The plasma samples analyzed in this study included: baseline, peak immunogenicity (2–4 weeks post last vaccination), and a durability time point (12 months post final). All groups were balanced for gender and age. All plasma samples were heat inactivated prior to experimental procedure at 56°C for 1h. Precipitate was spun down at 20,000g for 10 min and supernatant was stored at -80°C.

**Table 1:** Sample selection for HVTN study comparison.

TRIAL NAME	treatment group	age (mean)	gender		prime	boost	prime antigen	boost antigen
			male(%)	female(%)				
077	T1	34	0	100	Ad35 mo (0) (Gag/Pol/Nef/Env)	rAd5 mo (6) (gag/pol/nef/Env)	92RW020	92RW020
	T2	28	47	53	DNA mo (0,1,2) (gag/pol/nef/Env)	rAd5 mo (6) (gag/pol/nef/Env)	92RW020	92RW020
	T3	27	40	60	DNA mo (0,1,2) (gag/pol/nef/Env)	rAd35 mo (6) (gag/pol/nef/Env)	92RW020	92RW020
078	T1	23	0	100	NYVAC mo (0,1) (gag/pol/nef/Env)	rAd5 mo (6) (gag/pol/nef/Env)	BX08	92RW020, HXB2, 97ZA012
	T4	23	30	70	Ad5 mo (0) (gag/pol/nef/Env)	NYVAC mo (5,6) (gag/pol/nef/Env)	92RW020, HXB2, 97ZA012	BX08
105	T1	29	40	60	protein mo (0,1) (Env)	DNA mo (3,6) (gag/pol/nef/Env)	AIDSVAX B/E (MN/A244)	ZM96
	T2	28	33	67	DNA mo (0,1) (gag/pol/nef/Env)	protein mo (3,6) (Env)	ZM96	AIDSVAX B/E (MN/A244)
	T3	27	40	60	DNA mo (0,1,3,6) (gag/pol/nef/Env)	protein mo (3,6) (Env)	ZM96	AIDSVAX B/E (MN/A244)
	T4	26	67	33	DNA mo (0,1,3,6) (gag/pol/nef/Env)	protein mo (0,1,3,6) Env	ZM96	AIDSVAX B/E (MN/A244)
204	T1	30	47	53	DNA mo (0,1,2) (gag/pol/nef/Env)	rAd5 mo (6) (gag/pol/nef/Env)	92RW020, HXB2, 97ZA012	92RW020, HXB2, 97ZA012
205	T3	27	47	53	DNA mo (0,2) (gag/pol/nef/tat/rev/vpu/Env)	MVA mo (4,6) (gag/pol/Env)	HXB-2/ADA rec	HXB-2/ADA rec
	T4	27	53	47	MVA mo (0,2) (gag/pol/Env)	MVA mo (6) (gag/pol/Env)	HXB-2/ADA rec	HXB-2/ADA rec

### Antibody-dependent cellular phagocytosis (ADCP)

Monocyte mediated phagocytosis was assessed with a bead-based assay using the THP-1 cell line, as previously described [49]. Briefly, biotinylated (EZ-Link Sulfo-NHS-LC-LC-Biotin, Thermo Fisher) gp140 ConS (Duke University) antigen was coupled to 1μm yellow fluorescent neutravidin beads (Thermo Fisher) for 2 h at 37°C. After removal of excess antigen by washing with 0.1% Bovine serum albumin (BSA) in phosphate buffered saline (PBS) for blocking, saturated beads (1.82×10^8^ beads/well) were incubated with samples at a 1:100 dilution in PBS for 2 h at 37°C. Immune complexes were washed and 2.5×10^4^ THP-1 cells (American Type Culture Collection) were added per well and incubated for 16 h at 37°C. Cells were fixed in 4% paraformaldehyde (PFA) and sample acquisition was performed via flow cytometry (IntelliCyt, iQue Screener plus). Events were gated on single cells and bead positive cells. A phagocytosis score was calculated as the percent of bead positive cells× GMFI/1,000. All samples were run in duplicate on separate days.

### Antibody-dependent neutrophil phagocytosis (ADNP)

The antibody-dependent neutrophil phagocytosis assay was performed as previously described [50]. As described for the ADCP assay, neutravidin beads were saturated with biotinylated antigen gp140 ConS and incubated with 1:100 diluted plasma. Primary human PBMCs were isolated from healthy donors with Ammonium-Chloride-Potassium (ACK) lysis (Thermo fisher). PBMCs were washed and 5×10^4^ cells were added per well and incubated for 1 h at 37°C. Cells were stained with anti-CD66b Pac blue antibody (BioLegend) to identify CD66b positive neutrophils. After fixation with 4% PFA, cells were acquired via flow cytometry (IntelliCyt, iQue Screener plus). The neutrophil population was defined as CD66b+, and a phagocytosis score was calculated as described above. Data presented is the average of two different blood donors.

### Antibody-dependent complement deposition (ADCD)

Complement deposition was measured as described previously with a bead-based assay [51]. Briefly, biotinylated antigen gp140 ConS was coupled to red fluorescent neutravidin beads (Thermo fisher). Plasma samples were diluted 1:10 in PBS and allowed to form immune complexes with antigen coupled beads for 2 h at 37°C. Lyophilized guinea pig complement was resuspended according to manufacturer’s instructions (Cedarlane) and 4 μl per well was added in gelatin veronal buffer containing Mg^2+^ and Ca^2+^ (GVB^++^, Boston BioProducts) and incubated with the washed immune complexes for 20 min at 37°C. C3 deposition was detected with a fluorescein-conjugated goat IgG fraction to guinea pig complement C3 (MpBio). Complement coated beads were acquired via flow cytometry (IntelliCyt, iQue Screener plus) and C3 deposition was reported by gating on single beads and C3 positive events of two independent runs.

### Antibody-dependent NK cell degranulation (ADNKA)

Natural killer cell (NK) activation and degranulation via CD107a, IFN-γ and MIP-1β detection was assessed via an ELISA-based antibody-dependent natural killer (NK) cell activation assay [52]. ELISA plates (Thermo Fisher NUNC MaxiSorp flat bottom) were coated with gp140 ConS (200 ng per well) at 37°C for 2 h followed by blocking with 5% BSA in PBS overnight at 4°C. NK cells were isolated from buffy coats from healthy donors with RosetteSep (Stem Cell Technologies) and rested over night with 1 ng/ml IL-15. The next day, after washing the blocked ELISA plates, 50 μl of samples at a 1:25 dilution in PBS were added to each well. Plates were incubated at 37°C for 2 h to allow immune complex formation. Then, 5×10^4^ NK cells with anti-CD107a-PE-Cy5 stain (BD), brefeldin A (5 mg/ml) (Sigma), and GolgiStop (BD) were added to each well and incubated for 5 h at 37°C. NK cells were fixed and permeabilized using Perm A and B solutions (ThermoFisher). Cells were subsequently stained for surface markers with anti-CD16 APC-Cy7 (BD), anti-CD56 PE-Cy7 (BD) and anti-CD3 AlexaFluor 700 (BD). Intracellular staining included anti-IFNγ FITC (BD) and anti-MIP-1β PE (BD). Acquisition occurred by flow cytometry (IntelliCyt, iQue Screener plus). NK cells were defined as CD3- and CD56+. The antibody-dependent NK cell degranulation assay was performed in duplicate across two blood donors.

### Subclassing and isotyping via Luminex

A customized Luminex assay was used to quantify the relative concentration of antigen specific antibody subclass and isotype levels as well as Fcγ receptor (FcγR) binding [53]. Carboxyl-modified microspheres (Luminex) were coupled with HIV antigens (MN gp120, A244 gp120, HXB2 gp120, 92RW020 gp120, 97ZA012 gp140, 96ZM651 gp120, MN V1V2, Clade C V1V2, ConS gp140 and HXB2 gp41 (all Duke protein production facility) and CaseA2 V1V2 (Immunetech). Coupling was performed by covalent N-hydroxysuccinimide (NHS)- ester linkages via EDC (Thermo Scientific) and Sulfo-NHS (Thermo Scientific) according to the manufacturer’s instructions. 1.2×10^3^ beads per Luminex region per well were used in Luminex assay buffer containing 0.1% BSA and 0.05% Tween-20 and were added to each well of a 384-well plate (Greiner Bio-one). Plasma samples were diluted 1:50 in PBS for FcγR binding and IgG as well as IgA detection and were added and incubated for 16 h at 4°C and were rocked at 900 rpm. The microspheres were washed three times with 60 μl of Luminex assay buffer with an automated plate washer. PE-coupled IgG1, IgG3-, IgA1- and IgA2-specific detection reagents (Southern Biotech) were added and incubated with immune complexes for 1 h at room temperature while shaking at 900 rpm. The antigen-coated Luminex beads were then washed and read on a cytometer (IntelliCyt, iQue Screener Plus). Events were gated based on their bead fluorescence and PE MFI was analyzed as output. Each sample was run in duplicate in separate runs. Similarly, for the FcR binding profiles, recombinant FcγRIIA, FcγRIIB, FcγRIIIA, FcγRIIIB (Duke Protein Production facility) were biotinylated (Thermo Scientific), conjugated to Streptavidin- PE (Southern Biotech) before adding to immune complexes for 1 h. Samples were run in duplicate per each secondary detection agent.

### Statistics

Flow data was analyzed using the iQue IntelliCyt software. Statistical analysis and graphing were performed using GraphPad Prism 8, JMP V13 and Matlab. Spearman’s rank correlations were used to examine bivariate associations between variables. Bonferroni correction was applied as indicated in the Figure legends for multiple comparisons.

**Polyfunctionality** was calculated per functional readout: A given sample was considered to be functional for an assay if functionality above the median across all vaccinees for that function was induced. The number of functions each sample induced was summed to generate a polyfunctionality score ranging from a minimum of zero functions to a maximum of 6 functions for each sample.

**Correlation networks** were constructed for the top predictive features selected for polyfunctionality (Fig 5A) and ADCP durability (Fig 5B). Networks were based on the pairwise correlation coefficients between all measured biophysical and functional features per trial arm. This analysis was performed in MATLAB. Edges between nodes are weighted based on a significant correlation coefficient after correcting for multiple comparisons by Benjamini-Hochberg (BH). Networks show Spearman correlation coefficient > 0.7 and BH-adjusted p-value <0.01. Networks were generated using Cytoscape (Version 3.6.0).

**Radar plots** were generated for IgG1, IgG3, IgA1 and IgA2 relative titers against ConS gp140 in Fig 1B. Each antigen-specific response displayed was normalized to an average of 0 and a standard deviation of 1 across all trials, and the average of the subjects per group is graphed along the radars.

A **Principal Component Analysis (PCA)** model was constructed for the HVTN cohort using 69 antibody variables, including Fc-receptor measurements with non-zero median for each trial arm (Fig 4A). Variables were centered and scaled to a standard deviation of 1. Maximum separation was achieved in the two-dimensional space of PC1 (30.7%) vs. PC2 (18.5%). The PCA model was calculated and graphed in JMP (Version 13.2.0).

Due to the highly correlated nature of the antibody measurements, we used a combined **Least Absolute Shrinkage and Selection Operator (LASSO)** followed by **Partial Least Squares Discriminant Analysis (PLSDA)** to analyze multivariate profiles across vaccine arms/groups. LASSO was used to reduce dimensionality, only picking the features that contribute to the separation between groups along an ordinal dependent variable. Thus, features that are required to create a maximum separation are retained, but the model penalizes for the inclusion of additional features, aimed at only selecting a minimal feature set. The features are then visualized on the PLSDA plot. Samples are plotted using the scores in the first and the second latent variable (LV1 and LV2). To visualize the importance of each feature in LV1, the loading vectors are ranked by variable importance in projection (VIP) scores. VIP scores are the weighted sum of squares of the PLS weights, which summarize the importance of the various features to the PLSDA model. Model robustness was tested by performing 1000 repetitions of the PLSDA and a 10-fold cross validation. The model for separation of DNA/non-DNA trials resulted in 77.3% accuracy after ROC 1-fold cross validation with AUC of 0.74. Protein/non-protein comparison yielded 75.8% accuracy with 0.80 AUC after cross validation.

**Orthogonalized Partial Least Squares Regression (OPLSR)** was also used to mathematically identify the key features contributing to variation in polyfunctionality (Fig 5A) and durable ADCP responses (Fig 5C). This multivariate regression technique used linear combinations of features to predict the variance in either of the dependent variables. The model was then orthogonalized such that Latent Variable 1 (LV1) captured the variance in features that tracked in the direction of the dependent variable, while other latent variables described the variation orthogonal to the predictive component. Variables were z- scored and 5-fold cross validation was performed on the data (Venetian blinds) to test for model robustness. Additionally, model significance was assessed using permuted data by randomly shuffling the labels. For HVTN polyfunctionality at peak time point, 72 antibody variables were regressed onto polyfunctionality score, whereas for durability assessment, these same variables from peak time point were regressed onto ADCP phagoscores at month 12.

## Supporting information

S1 Raw Data All Systems Serology Data has been compiled in an excel file and is available as supporting material. Each row displays one sample, each column one measurement, labeled with assay name, followed by antigen. All Luminex (IgG1-4, IgA, IgM, FcRs) data is reported as MFI. ADCP and ADNP are reported as Phagoscore, ADCD as MFI of FITC (C3 deposition) and CD107a, MIP-1b and IFN-y as % of NK cell population.
(XLSX)

S1 Table Significance of pairwise comparison for Figs 1A and 2A. Kruskal Wallis test was performed, followed by Dunn’s correction for multiple comparisons. Adjusted P values are shown here.
(DOCX)
