# Supplementary material for: Sequence and vector shapes vaccine induced antibody effector functions in HIV vaccine trials
Source: PLoS Pathog. 2021 Nov 29;17(11):e1010016. doi: 10.1371/journal.ppat.1010016 (PMC8659322; doi:10.1371/journal.ppat.1010016)
Supplement: S1 Table — Kruskal Wallis test was performed, followed by Dunn’s correction for multiple comparisons. Adjusted P values are shown here. (DOCX) [file ppat.1010016.s002.docx]

**S1 Table:** Significance of pairwise comparison for Figs 1A and 2A. Kruskal Wallis test was performed, followed by Dunn’s correction for multiple comparisons. Adjusted P values are shown here.

| Comparisons between groups | IgG1 Adjusted P Value | IgG3 Adjusted P Value | IgA1 Adjusted P Value | IgA2 Adjusted P Value | ADCP Adjusted P Value | ADNP Adjusted P Value | ADCD Adjusted P Value | ADNKA CD107a Adjusted P Value | ADNKA IFN-y Adjusted P Value | ADNKA MIP-1b Adjusted P Value |
| --- | --- | --- | --- | --- | --- | --- | --- | --- | --- | --- |
| 77 T1 vs T2 | >0.9999 | 0.3049 | >0.9999 | >0.9999 | >0.9999 | >0.9999 | >0.9999 | 0.4803 | >0.9999 | >0.9999 |
| 77 T1 vs T3 | >0.9999 | >0.9999 | >0.9999 | >0.9999 | >0.9999 | >0.9999 | >0.9999 | 0.7705 | >0.9999 | >0.9999 |
| 77 T1 vs 78 T1 | >0.9999 | >0.9999 | 0.052 | >0.9999 | >0.9999 | 0.0911 | 0.2007 | 0.9997 | >0.9999 | >0.9999 |
| 77 T1 vs 78 T2 | >0.9999 | >0.9999 | >0.9999 | >0.9999 | >0.9999 | 0.0187 | 0.0203 | 0.9782 | >0.9999 | >0.9999 |
| 77 T1 vs 105 T1 | 0.4986 | 0.1132 | 0.4532 | <0.0001 | 0.0001 | <0.0001 | <0.0001 | 0.8245 | >0.9999 | 0.001 |
| 77 T1 vs 105 T2 | >0.9999 | >0.9999 | >0.9999 | 0.002 | >0.9999 | >0.9999 | >0.9999 | 0.9059 | >0.9999 | >0.9999 |
| 77 T1 vs 105 T3 | >0.9999 | >0.9999 | >0.9999 | 0.0297 | >0.9999 | >0.9999 | >0.9999 | 0.9183 | >0.9999 | >0.9999 |
| 77 T1 vs 105 T4 | >0.9999 | >0.9999 | >0.9999 | 0.0012 | >0.9999 | >0.9999 | >0.9999 | 0.8603 | >0.9999 | >0.9999 |
| 77 T1 vs 204 | >0.9999 | >0.9999 | >0.9999 | >0.9999 | >0.9999 | >0.9999 | >0.9999 | 0.6022 | >0.9999 | >0.9999 |
| 77 T1 vs 205 T3 | 0.7672 | >0.9999 | >0.9999 | 0.0083 | 0.1972 | 0.0008 | <0.0001 | 0.9094 | >0.9999 | 0.3261 |
| 77 T1 vs 205 T4 | >0.9999 | >0.9999 | >0.9999 | >0.9999 | >0.9999 | 0.0053 | 0.0025 | 0.9431 | >0.9999 | >0.9999 |
| 77 T2 vs 77 T3 | >0.9999 | >0.9999 | >0.9999 | >0.9999 | >0.9999 | >0.9999 | >0.9999 | >0.9999 | >0.9999 | >0.9999 |
| 77 T2 vs 78 T1 | >0.9999 | 0.5187 | 0.003 | >0.9999 | >0.9999 | 0.0162 | 0.0229 | 0.0144 | 0.2645 | 0.1213 |
| 77 T2 vs 78 T2 | >0.9999 | 0.016 | >0.9999 | >0.9999 | 0.9675 | 0.0031 | 0.0016 | 0.0058 | 0.0586 | 0.1178 |
| 77 T2 vs 105 T1 | 0.0435 | <0.0001 | 0.1013 | <0.0001 | <0.0001 | <0.0001 | <0.0001 | 0.0001 | 0.0502 | <0.0001 |
| 77 T2 vs 105 T2 | >0.9999 | 0.0138 | >0.9999 | 0.0003 | >0.9999 | >0.9999 | 0.3144 | 0.0004 | 0.0262 | 0.0035 |
| 77 T2 vs 105 T3 | >0.9999 | 0.1583 | >0.9999 | 0.0088 | >0.9999 | >0.9999 | >0.9999 | 0.0004 | 0.0538 | 0.0093 |
| 77 T2 vs 105 T4 | >0.9999 | <0.0001 | >0.9999 | 0.0001 | >0.9999 | >0.9999 | >0.9999 | 0.0002 | 0.0058 | >0.9999 |
| 77 T2 vs 204 | >0.9999 | >0.9999 | >0.9999 | >0.9999 | >0.9999 | >0.9999 | >0.9999 | >0.9999 | >0.9999 | >0.9999 |
| 77 T2 vs 205 T3 | 0.0822 | 0.0237 | >0.9999 | 0.0017 | 0.0223 | <0.0001 | <0.0001 | 0.0004 | 0.0147 | <0.0001 |
| 77 T2 vs 205 T4 | >0.9999 | 0.1685 | >0.9999 | >0.9999 | >0.9999 | 0.0003 | <0.0001 | 0.0007 | 0.0064 | 0.0185 |
| 77 T3 vs 78 T1 | >0.9999 | >0.9999 | 0.0078 | >0.9999 | >0.9999 | 0.2309 | 0.4292 | 0.0679 | 0.2429 | 0.4646 |
| 77 T3 vs 78 T2 | >0.9999 | 0.5705 | >0.9999 | >0.9999 | >0.9999 | 0.0446 | 0.0371 | 0.0269 | 0.0537 | 0.3959 |
| 77 T3 vs 105 T1 | 0.0199 | <0.0001 | 0.0448 | <0.0001 | <0.0001 | <0.0001 | <0.0001 | 0.0012 | 0.0457 | <0.0001 |
| 77 T3 vs 105 T2 | >0.9999 | 0.7292 | >0.9999 | 0.0015 | >0.9999 | >0.9999 | >0.9999 | 0.0028 | 0.0236 | 0.0196 |
| 77 T3 vs 105 T3 | >0.9999 | >0.9999 | >0.9999 | 0.0387 | >0.9999 | >0.9999 | >0.9999 | 0.0033 | 0.0488 | 0.0469 |
| 77 T3 vs 105 T4 | >0.9999 | 0.0036 | >0.9999 | 0.0008 | >0.9999 | >0.9999 | >0.9999 | 0.0017 | 0.0052 | >0.9999 |
| 77 T3 vs 204 | >0.9999 | >0.9999 | >0.9999 | >0.9999 | >0.9999 | >0.9999 | >0.9999 | >0.9999 | >0.9999 | >0.9999 |
| 77 T3 vs 205 T3 | 0.039 | >0.9999 | >0.9999 | 0.0084 | 0.0516 | 0.001 | <0.0001 | 0.0029 | 0.0132 | 0.0007 |
| 77 T3 vs 205 T4 | >0.9999 | >0.9999 | >0.9999 | >0.9999 | >0.9999 | 0.009 | 0.0026 | 0.0048 | 0.0058 | 0.0867 |
| 78 T1 vs 78 T2 | >0.9999 | >0.9999 | 0.021 | >0.9999 | >0.9999 | >0.9999 | >0.9999 | >0.9999 | >0.9999 | >0.9999 |
| 78 T1 vs 105 T1 | >0.9999 | 0.0008 | <0.0001 | <0.0001 | 0.0119 | >0.9999 | >0.9999 | 0.9898 | >0.9999 | 0.0301 |
| 78 T1 vs 105 T2 | >0.9999 | >0.9999 | 0.3225 | 0.2295 | >0.9999 | >0.9999 | >0.9999 | 0.9982 | >0.9999 | >0.9999 |
| 78 T1 vs 105 T3 | >0.9999 | >0.9999 | >0.9999 | >0.9999 | >0.9999 | >0.9999 | >0.9999 | 0.9988 | >0.9999 | >0.9999 |
| 78 T1 vs 105 T4 | >0.9999 | 0.9406 | 0.1519 | 0.1512 | >0.9999 | 0.293 | >0.9999 | 0.9946 | >0.9999 | >0.9999 |
| 78 T1 vs 204 | >0.9999 | >0.9999 | 0.0022 | >0.9999 | >0.9999 | 0.2045 | >0.9999 | 0.0279 | 0.1401 | 0.0623 |
| 78 T1 vs 205 T3 | >0.9999 | >0.9999 | <0.0001 | 0.786 | >0.9999 | >0.9999 | >0.9999 | 0.9984 | >0.9999 | >0.9999 |
| 78 T1 vs 205 T4 | >0.9999 | >0.9999 | <0.0001 | >0.9999 | >0.9999 | >0.9999 | >0.9999 | 0.9996 | >0.9999 | >0.9999 |
| 78 T2 vs 105 T1 | >0.9999 | 0.4174 | 0.2522 | 0.0053 | 0.4873 | >0.9999 | >0.9999 | >0.9999 | >0.9999 | 0.3391 |
| 78 T2 vs 105 T2 | >0.9999 | >0.9999 | >0.9999 | >0.9999 | >0.9999 | 0.2991 | >0.9999 | >0.9999 | >0.9999 | >0.9999 |
| 78 T2 vs 105 T3 | >0.9999 | >0.9999 | >0.9999 | >0.9999 | >0.9999 | 0.2991 | >0.9999 | >0.9999 | >0.9999 | >0.9999 |
| 78 T2 vs 105 T4 | >0.9999 | >0.9999 | >0.9999 | >0.9999 | >0.9999 | 0.0569 | >0.9999 | >0.9999 | >0.9999 | >0.9999 |
| 78 T2 vs 204 | >0.9999 | 0.3358 | >0.9999 | >0.9999 | >0.9999 | 0.0394 | 0.8056 | 0.0111 | 0.0309 | 0.0649 |
| 78 T2 vs 205 T3 | >0.9999 | >0.9999 | >0.9999 | >0.9999 | >0.9999 | >0.9999 | >0.9999 | >0.9999 | >0.9999 | >0.9999 |
| 78 T2 vs 205 T4 | >0.9999 | >0.9999 | >0.9999 | >0.9999 | >0.9999 | >0.9999 | >0.9999 | >0.9999 | >0.9999 | >0.9999 |
| 105 T1 vs105 T2 | >0.9999 | 0.0374 | 0.0006 | >0.9999 | 0.0102 | 0.001 | 0.1325 | >0.9999 | >0.9999 | 0.6476 |
| 105 T1 vs 105 T3 | 0.507 | 0.0025 | <0.0001 | 0.1095 | 0.0005 | 0.001 | 0.0115 | >0.9999 | >0.9999 | 0.3218 |
| 105 T1 vs 105 T4 | 0.1816 | >0.9999 | 0.0017 | >0.9999 | 0.0006 | <0.0001 | 0.0174 | >0.9999 | >0.9999 | 0.0011 |
| 105 T1 vs 204 | 0.1931 | <0.0001 | 0.1327 | <0.0001 | 0.0004 | <0.0001 | 0.0042 | 0.0004 | 0.025 | <0.0001 |
| 105 T1 vs 205 T3 | >0.9999 | 0.0221 | >0.9999 | 0.3895 | >0.9999 | >0.9999 | >0.9999 | >0.9999 | >0.9999 | >0.9999 |
| 105 T1 vs 205 T4 | >0.9999 | 0.0023 | >0.9999 | 0.0001 | 0.0213 | >0.9999 | >0.9999 | >0.9999 | >0.9999 | 0.1855 |
| 105 T2 vs 105 T3 | >0.9999 | >0.9999 | >0.9999 | >0.9999 | >0.9999 | >0.9999 | >0.9999 | >0.9999 | >0.9999 | >0.9999 |
| 105 T2 vs 105 T4 | >0.9999 | >0.9999 | >0.9999 | >0.9999 | >0.9999 | >0.9999 | >0.9999 | >0.9999 | >0.9999 | >0.9999 |
| 105 T2 vs 204 | >0.9999 | 0.4086 | >0.9999 | 0.0065 | >0.9999 | >0.9999 | >0.9999 | 0.0009 | 0.0123 | 0.0015 |
| 105 T2 vs 205 T3 | >0.9999 | >0.9999 | 0.1603 | >0.9999 | >0.9999 | 0.0142 | 0.1013 | >0.9999 | >0.9999 | >0.9999 |
| 105 T2 vs 205 T4 | >0.9999 | >0.9999 | >0.9999 | 0.9208 | >0.9999 | 0.095 | >0.9999 | >0.9999 | >0.9999 | >0.9999 |
| 105 T3 vs 105 T4 | >0.9999 | >0.9999 | >0.9999 | >0.9999 | >0.9999 | >0.9999 | >0.9999 | >0.9999 | >0.9999 | >0.9999 |
| 105 T3 vs 204 | >0.9999 | >0.9999 | >0.9999 | 0.1292 | >0.9999 | >0.9999 | >0.9999 | 0.001 | 0.0262 | 0.0042 |
| 105 T3 vs 205 T3 | 0.8471 | >0.9999 | 0.0368 | >0.9999 | >0.9999 | 0.0142 | 0.0084 | >0.9999 | >0.9999 | >0.9999 |
| 105 T3 vs 205 T4 | >0.9999 | >0.9999 | 0.5925 | >0.9999 | >0.9999 | 0.095 | 0.3294 | >0.9999 | >0.9999 | >0.9999 |
| 105 T4 vs 204 | >0.9999 | 0.0015 | >0.9999 | 0.0038 | >0.9999 | >0.9999 | >0.9999 | 0.0005 | 0.0025 | 0.786 |
| 105 T4 vs 205 T3 | 0.3201 | >0.9999 | 0.3434 | >0.9999 | >0.9999 | 0.0014 | 0.0128 | >0.9999 | >0.9999 | 0.8466 |
| 105 T4 vs 205 T4 | >0.9999 | >0.9999 | >0.9999 | 0.6406 | >0.9999 | 0.0122 | 0.4542 | >0.9999 | >0.9999 | >0.9999 |
| 204 vs 205 T3 | 0.3395 | 0.6192 | >0.9999 | 0.032 | >0.9999 | 0.0008 | 0.003 | 0.0009 | 0.0067 | <0.0001 |
| 204 vs 205 T4 | >0.9999 | >0.9999 | >0.9999 | >0.9999 | >0.9999 | 0.0078 | 0.1484 | 0.0015 | 0.0029 | 0.0086 |
| 205 T3 vs 205 T4 | >0.9999 | >0.9999 | >0.9999 | >0.9999 | >0.9999 | >0.9999 | >0.9999 | >0.9999 | >0.9999 | >0.9999 |
